# Exploring interspecies differences in ex vivo models of Pseudomonas aeruginosa keratitis: a comparative study of human, pig and sheep corneas

**DOI:** 10.1099/jmm.0.001901

**Published:** 2024-12-13

**Authors:** Katarzyna Okurowska, Sheila MacNeil, Sanhita Roy, Prashant Garg, Peter N. Monk, Esther Karunakaran

**Affiliations:** 1 Department of Health Sciences, University of York, York, UK; 2 Department of Materials Science and Engineering, University of Sheffield, Sheffield, UK; 3 Prof. Brien Holden Eye Research Centre, LV Prasad Eye Institute, Hyderabad, India; 4 The Cornea Institute, LV Prasad Eye Institute, Hyderabad, India; 5 Department of Infection, Immunity and Cardiovascular Disease, University of Sheffield, Sheffield, UK

**Keywords:** *ex vivo* human cornea, *ex vivo* pig cornea, *ex vivo* sheep cornea, keratitis, *P. aeruginosa*

## Abstract

**Introduction.** Interspecies differences in human, pig and sheep corneal thickness may affect the *Pseudomonas aeruginosa* colonization. Currently, there is no research investigating the impact of these differences, along with variable storage and culture conditions on infection in *ex vivo* cornea models. These factors could significantly influence utilizing *ex vivo* models for drug testing research.

**Aim.** In this study, we aim to compare the relevance of sheep and pig cornea infection models to human.

**Methodology.** The corneas were stored in McCarey-Kaufman medium or Eagle’s Minimum Essential Medium or Dulbecco’s Modified Eagle’s Medium/Mixture F-12 Ham medium (incubator) and then infected after varying storage durations. The effect of added foetal bovine serum (FBS) to media and continuous shaking mimicking rinsing with tears on infection was also investigated. The infection outcome was evaluated by comparing c.f.u. between conditions.

**Results.** The study found that storage conditions, culture media, FBS and continuous rinsing of corneas with media had no significant effect on infection progression in *ex vivo* keratitis models across selected species.

**Conclusions.** Pig and sheep models yield results comparable to human corneas. These findings support the interchangeability of *ex vivo* human, pig and sheep keratitis models for *P. aeruginosa* infection studies, emphasizing their relevance and reliability in research contexts. This interchangeability is particularly useful for research groups where one particular animal model may not be available. The media in this *ex vivo* keratitis model can be free of animal components by the removal of FBS, which reduces the reliance on animal-derived products, aligning with ethical considerations and promoting more sustainable and humane scientific practices. This study advances the understanding of *ex vivo* keratitis models, demonstrating their robustness and potential for broader application in ophthalmic research and drug testing.

## Data Summary

The authors confirm that all supporting data and protocols have been provided in the article.

## Introduction

The cornea is the transparent front of the eye that allows passing the light through. Various factors can lead to corneal injury, increasing the risk of infection (keratitis). Globally, 1.5–2.0 million cases of unilateral blindness are caused by microbial keratitis annually [1]. There are a few types of bacteria that can cause keratitis, but one frequently found in patients is *Pseudomonas aeruginosa* [2]. Keratitis induced by *P. aeruginosa* progresses rapidly, posing significant challenges to treatment, particularly in developing countries [2]. The consequential damage often results in compromised vision or even the loss of an eye, affecting both humans and domesticated animals. Finding new treatments is challenging because many drugs toxic to bacteria are also harmful to humans [3,4]. Consequently, the success rate of drug development, including those targeting keratitis, among other conditions, stands at a mere 5%, with failures predominantly occurring before clinical trials [5].

Traditionally, the safety assessment of novel drugs relies on testing in live animal models before progressing to clinical trials. Using live animals for experiments is expensive and requires specific facilities and training, often rendering it inaccessible to many research groups. *Ex vivo* cornea models could be a promising and cost-effective addition for investigating infection dynamics and conducting preliminary evaluations of new antimicrobials before the *in vivo* testing. However, the lack of standardized protocols and limited clarity regarding their reproducibility and translatability to human relevance needs clarification [6]. Hence, our aim is to establish standardized protocols, enhancing clarity, data quality and reproducibility in *ex vivo* keratitis models.

In the literature, research groups use corneas from various species, differing media compositions and experimental setups [7,9], complicating cross-study comparisons. Encountering challenges in procuring high-quality rabbit corneas, we developed a reproducible *ex vivo* pig keratitis model in previous studies [10,11]. In these studies, we optimized the rinsing of corneas to remove detached bacteria, inoculum size, wounding techniques and culture conditions. The glass mould confines the bacteria to a 10-mm diameter area on top of the stratified squamous epithelium. Our optimizations led to reproducible and predictable bacterial counts from infected *ex vivo* pig and rabbit corneas. In our first study [11], we showed that there was no difference in c.f.u. between rabbit and pig corneas *ex vivo* infected with *P. aeruginosa*. Additionally, in our recent publication, we demonstrated an increase in bacterial growth in *ex vivo* porcine corneas infected with *P. aeruginosa* PA01 over 24 h [10]. Our *ex vivo* pig and sheep corneas are sourced from animals sacrificed for human consumption and otherwise would be discarded. Pig corneas were easier to obtain and exhibited greater consistency in quality, animal age and breed compared with rabbits.

In this study, we aim to compare the relevance of sheep and pig cornea models to human keratitis. Notably, while tissue banks worldwide use varied storage and maintenance methods for human corneas, these protocols primarily focus on suitability for transplantation rather than infection studies. Addressing this gap, we also obtained corneas from diverse tissue banks, replicating their maintenance conditions in our porcine cornea model to assess their impact on infection progression. Additionally, we investigated the effect of dextran, used in an Indian cornea bank to prevent swelling, on infection outcomes. As the sole animal-derived component in our media, we assessed the effect of foetal bovine serum (FBS) addition on infection, aiming to develop animal component-free media in adherence to the 3Rs principle [12]. Furthermore, by including sheep corneas, readily obtainable from abattoirs, we expand the scope of our investigation. In addition, we conducted experiments simulating frequent rinsing of corneas to mimic tears, aiming to elucidate its potential role in the infection progression. Our overarching goal remains the standardization and refinement of experimental conditions, ensuring the production of reliable models for studying keratitis and facilitating data comparison across studies and species.

## Methods

### Bacterial strain used

We infected all *ex vivo* corneas in this study with *P. aeruginosa* strain PA01. The strain was a gift from Professor Urs Jenal, University of Basel, Switzerland.

### Human corneas

We collected the corneas from human eye banks in the UK and India. In the UK, consent was obtained from donors in agreement with the tenets of the Declaration of Helsinki, including families’ consent for clinical and scientific use, and then, these were stored in the tissue bank of the National Health Service Blood and Transplant (NHSBT) established under SI 2005 No. 2529, UK. The corneas used for the experiments described here were kept for no longer than a month and were rejected for transplantation because of low endothelial density. The Human Research Authority and the University of Sheffield Ethics Committee (application no. 032473) approved the study. The NHSBT did not provide details of individual donors. Additionally, the corneas used in experiments were randomly assigned, to minimize selection bias and the impact of unknown factors that could influence the outcomes. The tissue bank supplied the corneas in Eagle’s Minimum Essential Medium (EMEM) with Earle’s salts supplemented with 2% foetal calf serum, 100 U ml^−1^ penicillin, 0.1 mg ml^−1^ streptomycin and 0.25 µg ml^−1^ amphotericin B. The corneas were stored at 34–36 °C before being dispatched to our laboratory.

We also studied infection in a batch of human cadaveric corneas acquired from the Ramayamma International Eye Bank, LV Prasad Eye Institute (LVPEI), Hyderabad, India. These corneas were stored in tissue culture media 199 [McCarey-Kaufman (MK) media] buffered with 5.95 g l^−1^ HEPES and 1.75 g l^−1^ NaHCO_3_ for 6 to 22 days at 4 °C. The media were supplemented with 50 g l^−1^ dextran (mw. 40K) and 0.1 g l^−1^ gentamicin. Media and all the components were purchased from Sigma (Germany).

### Pig corneas

Pig eyes were obtained from pigs sacrificed for human consumption and not for this study; therefore, ethical approval was not required. The pigs used in this study were white landrace sows, and the breed was a cross with a Hampshire boar. The age of pigs varied between 21 and 23 weeks. The eyes were harvested within 4 h from slaughter and transported from the abattoir (RP Elliott Abattoir, Calow, England) in a Nalgene container filled with sterile PBS (Sigma, Germany). The extraction, incubation and infection procedures were followed as published previously [11]. The excised corneas were incubated in a combination of Dulbecco’s Modified Eagle’s Medium (DMEM) and Ham’s F12 Nutrient Mixture (1 : 1) (Sigma, Germany) supplemented with 5 μg ml^−1^ insulin (SLS, UK), 10 ng ml^−1^ epidermal growth factor (EGF) (SLS, UK) and 10% foetal calf serum (FCS) (optional) (Pan-Biotech, UK) in a 6-well plate for 24 h [11]. Following this, the corneas were washed twice with 1 ml PBS and incubated in an antimicrobial-free DMEM: Ham’s F12 media for 48 h to remove residual antibiotics from the previous media. Throughout this time, the medium was replaced daily. We confirmed the absence of bacterial growth inhibition after 48 h in media without antibiotics (unpublished data). Additionally, we regularly inspected corneas for contamination by processing control (uninfected) corneas and incubating a small sample of media from each cornea on LB agar just before infection experiments.

A small batch of pig corneas was stored at 4 °C for 1 week in the same MK media composition as the one used for culturing human corneas in the LVPEI. Three pig corneas were incubated at 37 °C on a rocker (Fisher Scientific, USA) to mimic the blinking until the infection day. The experiment was repeated three times. The corneas were rocked eight times per minute at a 6-degree angle as described in the literature [13].

### Sheep corneas

Sheep eyes were obtained the same way as pig eyes, except that we extracted the eyes from sheep heads in our laboratory. We cleaned the extracted corneas and cultured them at 37 °C as described previously [11]. Sheep corneas were cultured as pig eyes and infected after 3 days from harvesting.

### Infection of *ex vivo* corneas

*P. aeruginosa* PA01 was seeded on LB agar and incubated at 37 °C overnight. On the day of the investigation, the bacteria from the agar plate were sub-cultured into LB broth and incubated for 2–3 h at 37 °C. We prepared the inoculum as described previously [11].

All corneas were removed from the existing media (Table 1) and processed as described in our standard procedure [11]. The corneas were wounded and infected with an average of 6×10^6^ c.f.u. of *P*. *aeruginosa*, exactly as described [11]. Each batch of the corneas included an uninfected control cornea (sterility control) immersed in 0.2 ml of PBS, along with the corneas infected with *P. aeruginosa* PA01 suspended in PBS. The number of replicates per batch varied depending on cornea availability. Typically, a batch of sheep corneas comprised one control cornea and two infected corneas. Since more porcine corneas were available, these batches contained more replicates. Each experiment was repeated at least three times. Human corneas were received in small quantities, sometimes only two or three at a time, so experiments were repeated whenever the tissue was available. All corneas were rinsed twice before homogenization and plating with 1 ml of PBS to remove non-adherent bacteria [11]. The control corneas were also rinsed, homogenized and plated. No growth was observed on agar plates from the control corneas. We calculated c.f.u. from infected corneas after 6 and 24 h of incubation at 37 °C.

**Table 1.** Differences in culture conditions between *ex vivo* cornea models before infection experiments

Culture conditions	*Ex vivo* cornea models			
	Human NHSBT	Human LVPEI	Pig	Sheep
MK media		+	+	
EMEM media	+			
Dextran		+		
EGF			+	+
Insulin			+	+
FBS	+		+	+
Penicillin	+		+	+
Streptomycin	+		+	+
Amphotericin B	+		+	+
Gentamicin		+	+	
Temp. 34–37 °C	+		+	+
Temp. 4 °C		+	+	

All corneas were photographed with a Dino-lite Xcope camera (AnMo Electronics Corporation, Taiwan).

‘+’ indicates that the component or condition was included in the setup.

### Histology

Immediately before and after infection with PA01 for 24 h, *ex vivo* porcine corneas were placed in 4% paraformaldehyde for 30 min. The corneas were dehydrated in isopropanol series and waxed in tissue processor TP1020. Waxed tissue was sliced to a thickness of 10 µm with Microtome Leica RM2145 in the central part and fixed on a microscopy slide at 37 °C using a heating plate for 20 min. The corneas were rehydrated by dipping in 100% xylene twice for 2 min and then 100% isopropanol for 2 min, followed by 95% (v/v) and 80% (v/v) isopropanol for 1 min. The corneas were rinsed with distilled water for 2 min before staining with haematoxylin and eosin (H&E) to evaluate tissue morphology and Gram staining to visualize bacteria. Slides with cornea sections were immersed in haematoxylin for 4 min, followed by rinsing under tap water for 3 min. Next, the corneas were immersed in a bluing solution for 1 min and then rinsed with tap water again for 1 min. The next step involved dipping slides with the corneas in eosin for 1 min, followed by rinsing with 95% isopropanol for 15 s, 100% isopropanol for 30 s and, finally, with xylene for 2 min.

A few slides with tissue sections were designated for Gram staining to visualize bacteria, and the kit (Pro-Lab Diagnostics, UK) with already prepared solutions was used. First, the tissue sections were covered with Crystal violet for 60 s and then immediately rinsed with tap water. In the next step, the section was covered with iodide for 30 s, which binds to Crystal violet and traps it in the cell. After removing the dye with tap water, the tissue section was rinsed quickly with decolorizing solution (about 5 s) and then counterstained with safranin for 60 s. After rinsing with tap water, the tissue section was dried with blotting paper. The slide was mounted with a mounting media (Sigma, Germany) and covered with a cover slip. All histological sections were imaged using an upright microscope Olympus BX51 (Essex, UK) and the software ProgRes Capture Pro 2.5 (Jenoptik).

### Statistics

Statistical analysis comparing viable cell count of *P. aeruginosa* between *ex vivo* human, pig and sheep keratitis models was carried out using a Mann–Whitney test using GraphPad Prism version 8.4.1. *P*-values lower than 0.05 were considered significant.

## Results

### Cornea images

**Fig. 1.**
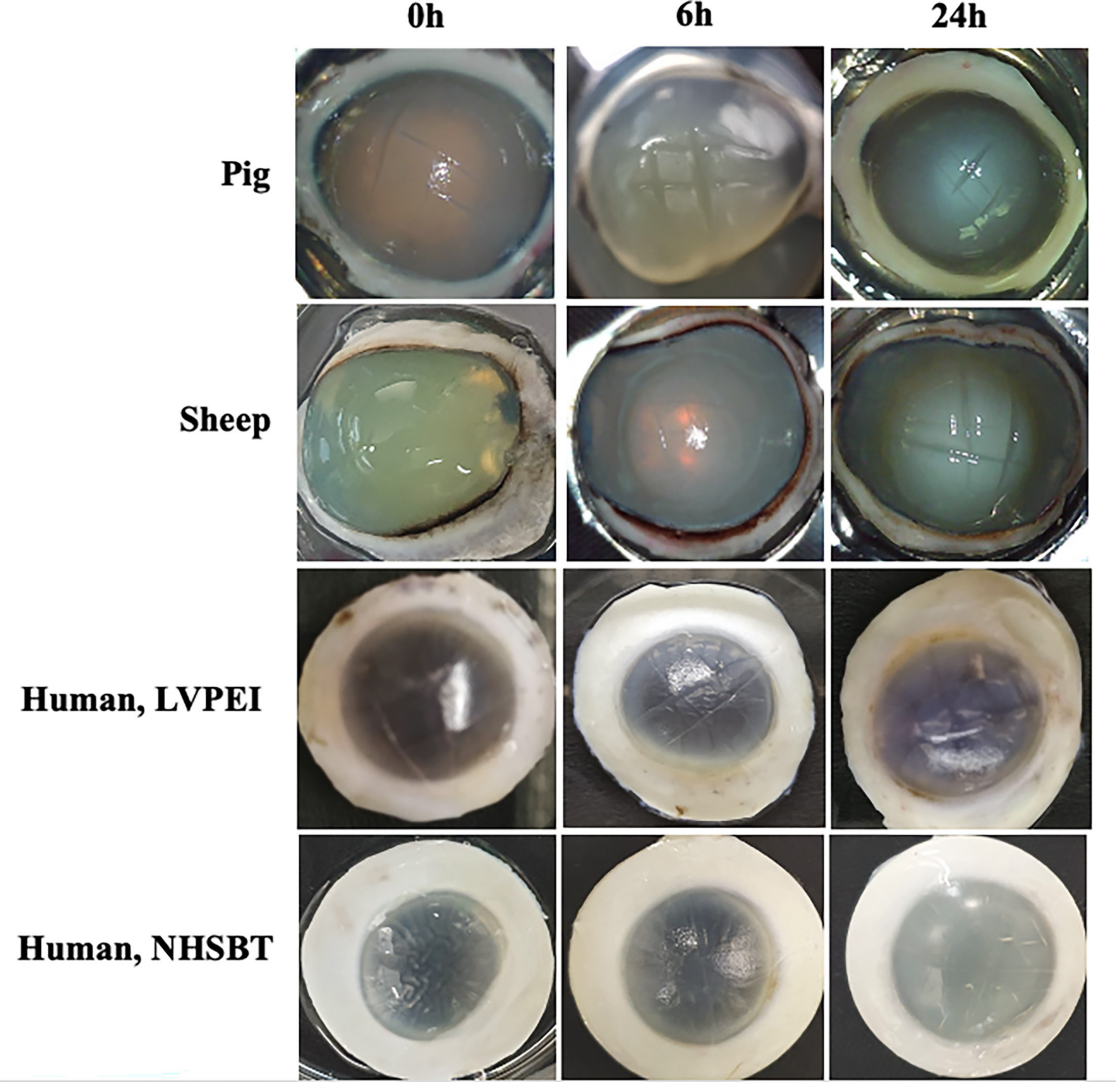
Representative images of non-infected (0 h) and infected *ex vivo* pig, sheep and human corneas. The corneas shown here were infected with 6×10^6^ c.f.u. of *P. aeruginosa* PA01 for 6 and 24 h.

All corneas were photographed before homogenization. The images captured uninfected (0 h) pig, sheep and human corneas, as well as those infected with *P. aeruginosa* PA01 for 6 and 24 h (Fig. 1). *Ex vivo* corneas often swell slightly when kept in media for several days, resulting in a hazy appearance, as previously reported [10,11]. After 6 and 24 h of incubation with bacteria, more swelling and white discolouration developed, with cuts in the central part of the cornea becoming more visible at 24 h. Human corneas swelled less than pig and sheep corneas, possibly due to the presence of Bowman’s layer [14], which is thinner or absent in other animals [15,16]. Human corneas retained their opaque shape without support, while pig and sheep corneas required agarose support for imaging to prevent collapse, making them visually less transparent than the human corneas (Fig. 1). Visually, there was no difference between human corneas from the LVPEI and NHSBT despite variations in storage media, temperature and duration. The observed white discolouration and increased swelling compared with uninfected controls suggest that bacterial activity leads to corneal damage.

**Fig. 2.**
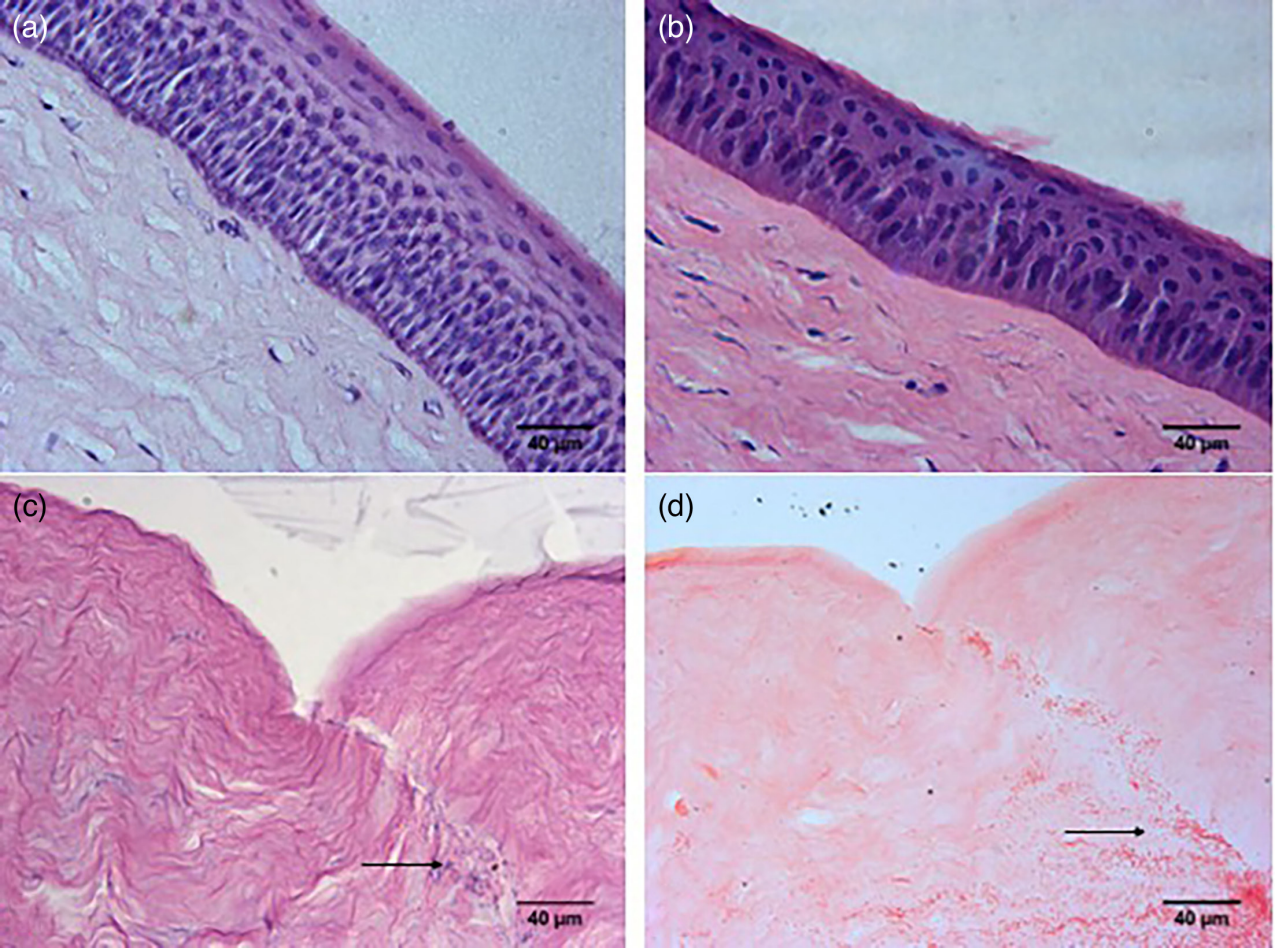
Histology of porcine corneas before and after infection with *P. aeruginosa*. Images of (**a**) uninfected porcine cornea immediately after enucleation, (**b**) before infection and (**c**) after infection with PA01 for 24 h (H&E). (**d**) Porcine cornea infected with PA01 for 24 h (Gram-stained). Arrows point at the bacteria inside the cut in the stroma. The corneas were processed in duplicate, and representative samples are shown here.

### Histology

Paraffin-fixed embedded tissue sections stained with H&E (Figs1b, c  2a) and Gram-stained (GS) (Fig. 1d) to localize bacteria in the cornea. Haematoxylin stains cell nuclei blue, while eosin stains the extracellular matrix and cytoplasm pink. H&E stain visualizes cells’ general layout and distribution and shows tissue structure. Fig. 1a shows intact, healthy, well-defined stratified squamous epithelium at the top and stroma with sparsely located keratocytes in the porcine cornea immediately after enucleation. The stratified squamous epithelium is visible along with a part of the stroma with a few keratocytes underneath. After 3–4 days in media during the preparation of the cornea and just before infection, the stratified squamous epithelium looked thinner, but still, it was present and intact across the corneal surface (Fig. 2b). After 24 h of infection, the stratified squamous epithelium was no longer visible in the central part of the cornea that had been exposed to PA01 (Fig. 2c and d). The collagen fibres in the superficial stroma looked disorganized, and keratocytes were no longer visible in the top part of the stroma. The cut area in the stroma was filled with clumps of *P. aeruginosa*, indicating that bacteria were proliferating there. H&E stained bacteria dark blue (Fig. 2c), while Gram stain shows them in dark red (Fig. 2d).

### Effect of different media and culture conditions on infection in human corneas

**Fig. 3.**
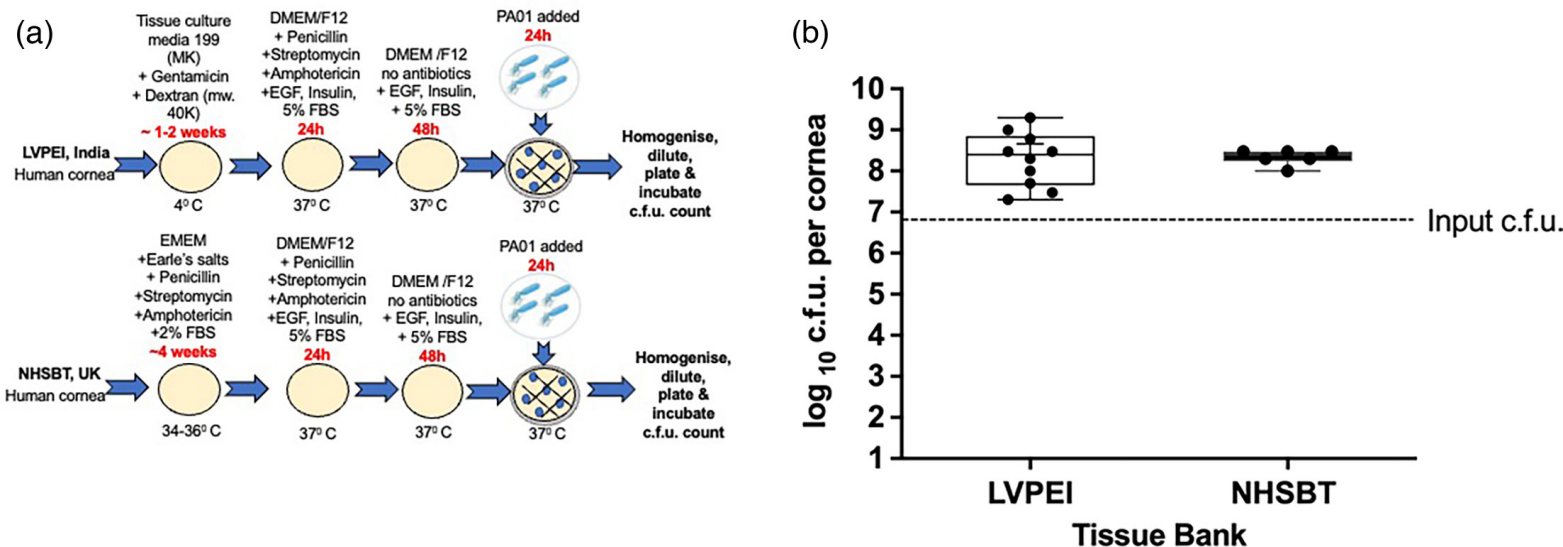
Comparison of c.f.u. in the corneas from the LVPEI and NHSBT tissue banks. A schematic image of culture conditions (**a**) and a graph comparing c.f.u. for *ex vivo* human corneas infected with *P. aeruginosa* PA01 for 24 h (**b**). Human corneas from the LVPEI were stored in MK media in the fridge for 1–2 weeks before the experiment. Human corneas from the NHSBT were incubated in EMEM media for up to 1 month before the investigation. The box plot shows the minimum and maximum values depicted by the bars, and the top, middle and bottom horizontal lines show the upper, median and lower quartiles. Data with a *P*-value>0.05 were not significant. Statistical significance was calculated with the Mann–Whitney test, *P*-value>0.05.

To establish if different types of media and culture conditions affect the progress of the infection, we infected human corneas obtained from two tissue banks (LVPEI and NHSBT) and stored them under different conditions. The LVPEI provided human corneas in MK media (*n*=10) held in the fridge before experiments (Fig. 3a). The NHSBT (UK) (*n*=6) kept human corneas in EMEM media and then delivered them to our laboratory for experiments (Fig. 3a). We transferred all human corneas to DMEM/F12 media without antibiotics for 2 days. After this time, we infected the corneas with 6×10^6^ of *P. aeruginosa* PA01 for 24 h. We retrieved an average of 5×10^8^±6×10^8^ colonies from *ex vivo* human corneas obtained from the LVPEI and 2×10^8^±8×10^7^ from the corneas from the NHSBT (Fig. 3b) after overnight incubation. The Mann-Whitney test showed no significant difference (*P*>0.5) in c.f.u. for human corneas coming from these two tissue banks. Different media composition and antibiotics during storage, adding dextran to MK media during storage, storage temperature, length of storage and variations between donors, did not affect the infection outcome in human corneas infected with *P. aeruginosa* PA01 for 24 h.

### Effect of different media and culture conditions on infection in pig corneas

**Fig. 4.**
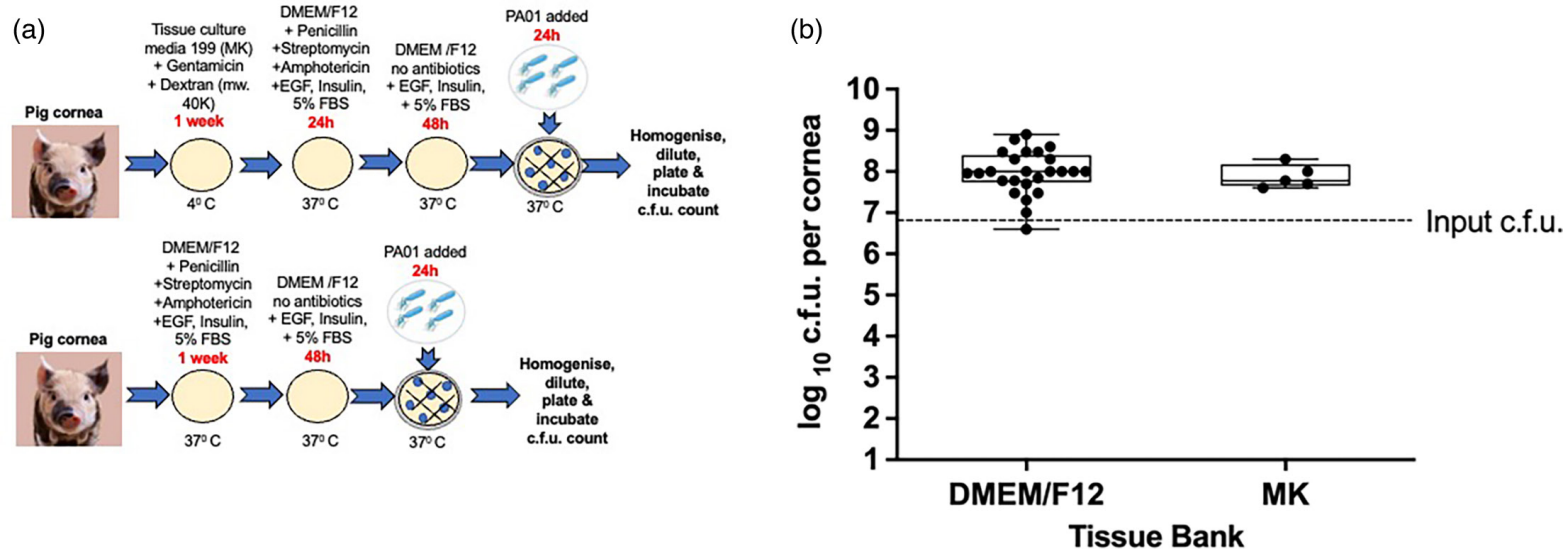
Effect of media on c.f.u. in pig corneas. A schematic image of culture conditions (**a**) and a graph comparing c.f.u. for *ex vivo* pig corneas infected with *P. aeruginosa* PA01 for 24 h (**b**). One batch of pig corneas was stored in MK media with dextran in the fridge before the experiment. The second batch was stored in DMEM/F12 media in the incubator. The box plot shows the minimum and maximum values depicted by the bars. The top, middle and bottom horizontal lines demonstrate the upper, median and lower quartiles. Statistical significance was calculated with the Mann–Whitney test, *P*-value>0.05.

To see if a variation in storage conditions in human corneas would have the same effect on pig corneas, we kept pig corneas in MK media with dextran (*n*=5) in the fridge for a week before proceeding with our standard protocol [11]. The MK media had no insulin, EGF and FBS added (Fig. 4a). We processed the other batch of *ex vivo* pig corneas cultured in DMEM/F12 at 37 °C (*n*=25) within 5 days from extraction (Fig. 4a). We infected all *ex vivo* pig corneas in this experiment with *P. aeruginosa* for 24 h (Fig. 4b). On an average, 9×10^7^±7×10^7^ c.f.u. was retrieved from pig corneas stored in MK media and 2×10^8^±2×10^8^ c.f.u. from the corneas cultured in DMEM/F12. The outcome of 24 h infection with *P. aeruginosa* PA01 in *ex vivo* pig corneas was very similar (*P*>0.5) regardless of the media, antibiotics, growth supplements or the storage temperature before experiments.

### Comparing infection outcomes between humans, pigs and sheep corneas

**Fig. 5.**
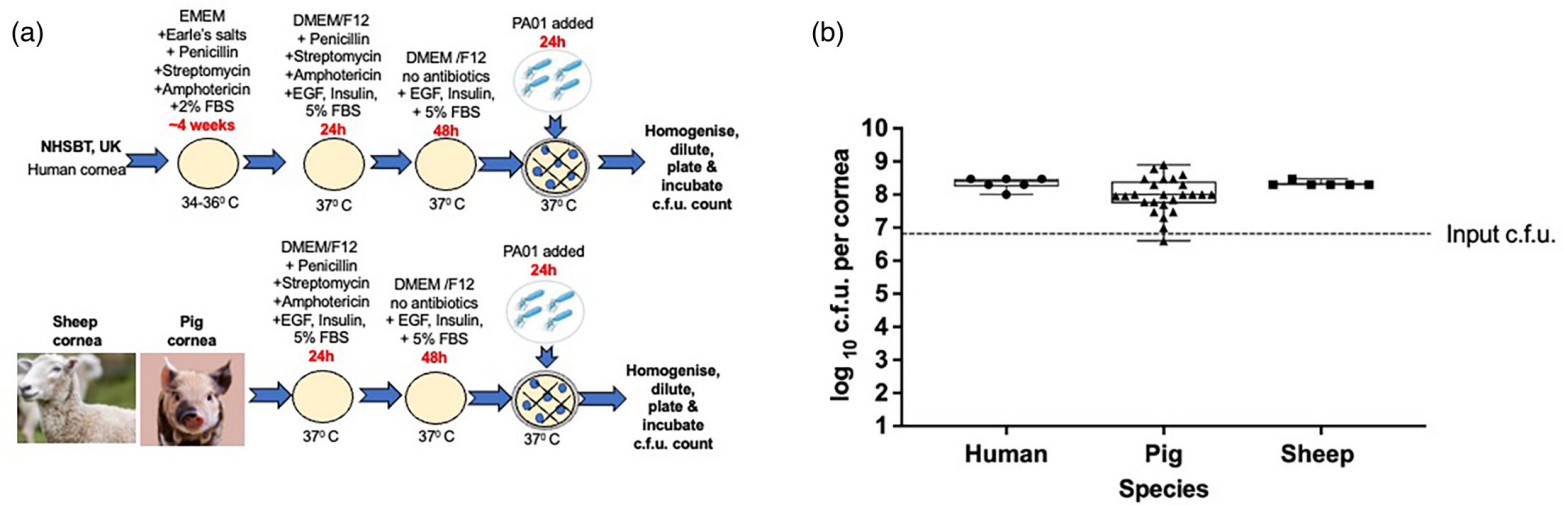
Comparison of c.f.u. in the corneas from human, pig and sheep. A schematic image of culture conditions (**a**) and a graph comparing c.f.u. for *ex vivo* human, pig and sheep corneas infected with *P. aeruginosa* PA01 for 24 h (**b**). The box plot shows the minimum and maximum values depicted by the bars. The top, middle and bottom horizontal lines demonstrate the upper, median and lower quartiles. Statistical significance was calculated with the Mann–Whitney test, *P*-value>0.05.

Finally, we compared infection between *ex vivo* human (NHSBT) (*n*=6), pig (*n*=25) and sheep (*n*=6) cornea models (Fig. 5b) infected with *P. aeruginosa* PA01 for 24 h. Most of the corneas were cultured in DMEM/F12 at 36–37 °C except for human corneas that were stored in EMEM media (Fig. 5a). The average c.f.u. for *P. aeruginosa* PA01 retrieved from *ex vivo* corneas were the following: human 2×10^8^±8×10^7^, pig 2×10^8^±2×10^8^ and sheep 2×10^8^±4×10^7^ (Fig. 5b). After 24-h infection, the c.f.u. was significantly higher than the input (*P*<0.0001 for human, pig and sheep). Interspecies differences did not affect the infection outcome after 24 h of incubation with *P. aeruginosa*. Similar infection outcomes for all animal and human corneas suggest that *ex vivo* pig and sheep cornea models could be an alternative for studying infection in human corneas.

**Fig. 6.**
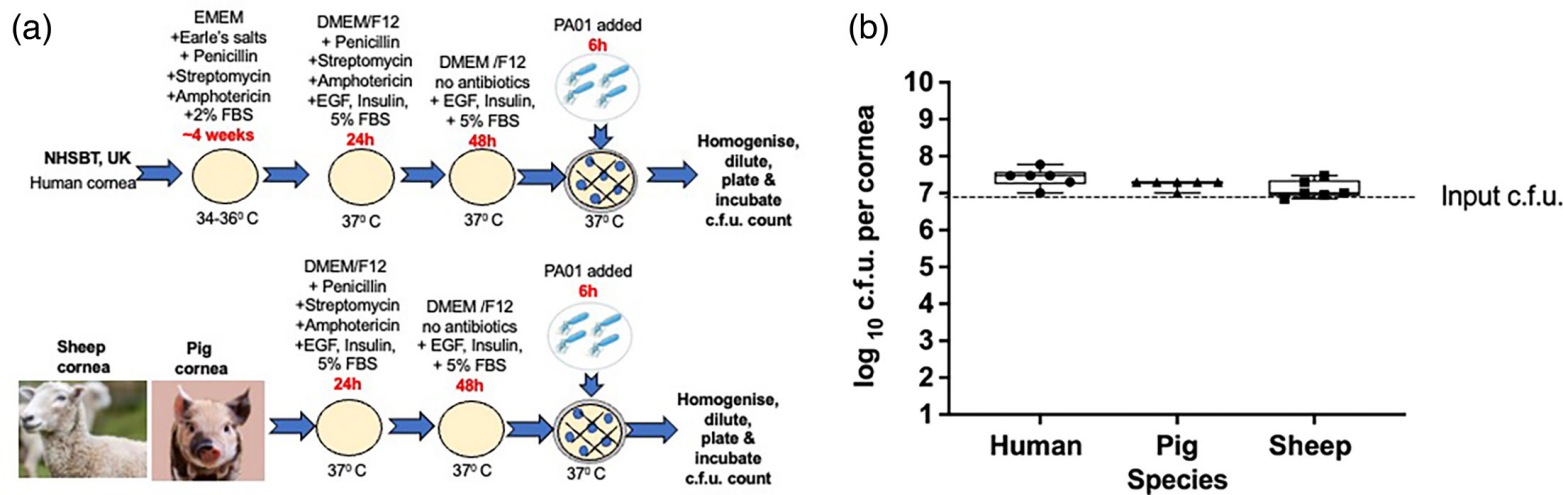
A schematic image of culture conditions (**a**) and a graph comparing c.f.u. for *ex vivo* human, pig and sheep corneas infected with *P. aeruginosa* PA01 for 6 h (**b**). Culturing conditions were the same for all the corneas. The box plot shows the minimum and maximum values depicted by the bars. The box plot shows the minimum and maximum values depicted by the bars. The top, middle and bottom horizontal lines demonstrate the upper, median and lower quartiles. Statistical significance was calculated with the Mann–Whitney test, *P*-value>0.05.

After 24 h, the infection was advanced because the bacteria load was much higher than the initial input (6×10^6^ c.f.u.s), indicating that *P. aeruginosa* successfully attached and proliferated on the tissue. To investigate if the results would be similar at earlier stages of infection, we infected *ex vivo* human (*n*=6), pig (*n*=6) and sheep (*n*=6) corneas with *P. aeruginosa* PA01 for 6 h (Fig. 6b). Pig and sheep corneas were incubated in DMEM/F12 with antibiotics, while human corneas were incubated in EMEM medium at 37 °C before experiments (Fig. 6a). Average colony counts were as follows: human 3×10^7^±2×10^7^, pig 2×10^7^±4×10^6^ and sheep 2×10^7^±9×10^6^ (Fig. 6b). Again, this shorter incubation time resulted in higher bacterial load than input and similar infection outcomes between *ex vivo* human, pig and sheep corneas infected with *P. aeruginosa* PA01. These results also suggest that interspecies differences have no significant impact during the early stages of infection with *P. aeruginosa* (*P*>0.5).

### Effect of media supplementation with FBS

**Fig. 7.**
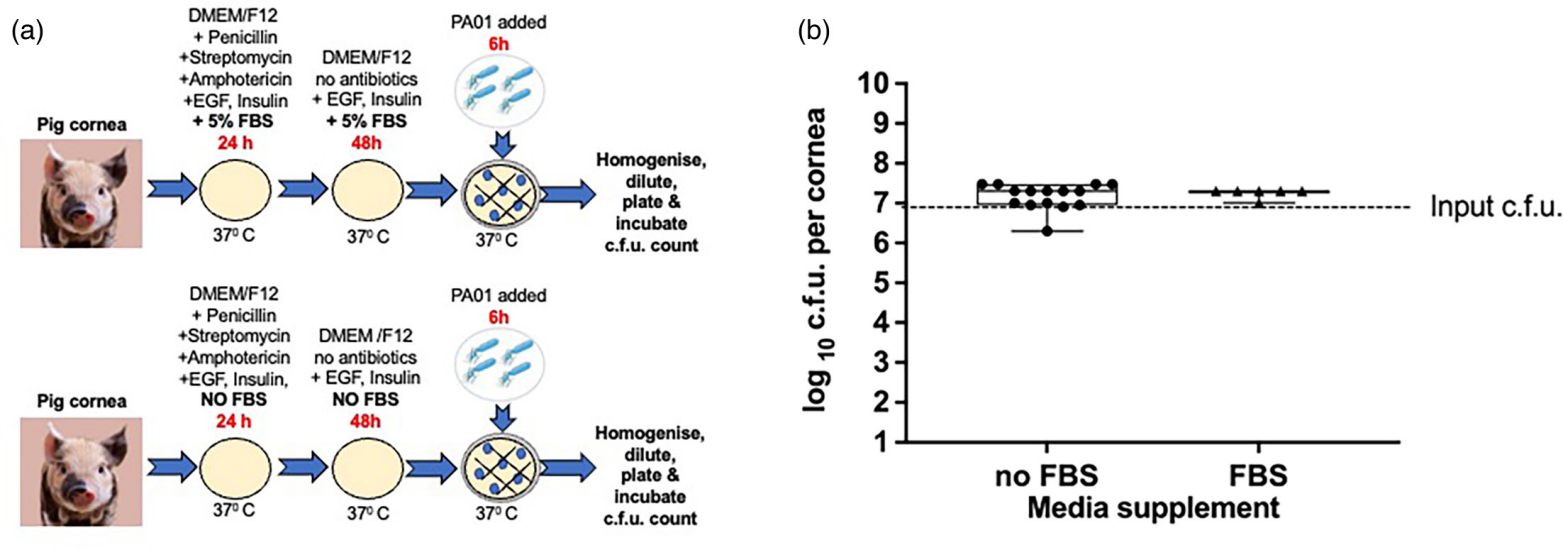
Effect of FBS on infection progression. Schematic image of culture conditions (**a**) and a graph comparing c.f.u. for *ex vivo* pig corneas incubated in DMEM/F12 media with and without the addition of FBS (**b**). Corneas were infected with *P. aeruginosa* PA01 for 6 h. The box plot shows the minimum and maximum values depicted by the bars. The top, middle and bottom horizontal lines demonstrate the upper, median and lower quartiles. Statistical significance was calculated with the Mann–Whitney test, where a *P*-value<0.05 shows significance.

We investigated the impact of adding FBS on the progression of infection because we aim to eliminate the reliance on animal-derived components in our experimental setup. Therefore, *ex vivo* pig corneas were cultured in DMEM/F12 media at 37 °C with (*n*=9) and without (*n*=9) FBS added (Fig. 7a, b). We infected the corneas with *P. aeruginosa* PA01 for 6 h, and the viable bacteria were calculated (Fig. 7b). The outcome of the infection was the same regardless of supplementation with FBS. Average colony counts were as follows: FBS 2×10^7^±4×10^6^, no FBS 2×10^7^±9×10^6^. There was no significant difference between these two conditions (*P*>0.05).

### Effect of rocking corneas on infection progression

**Fig. 8.**
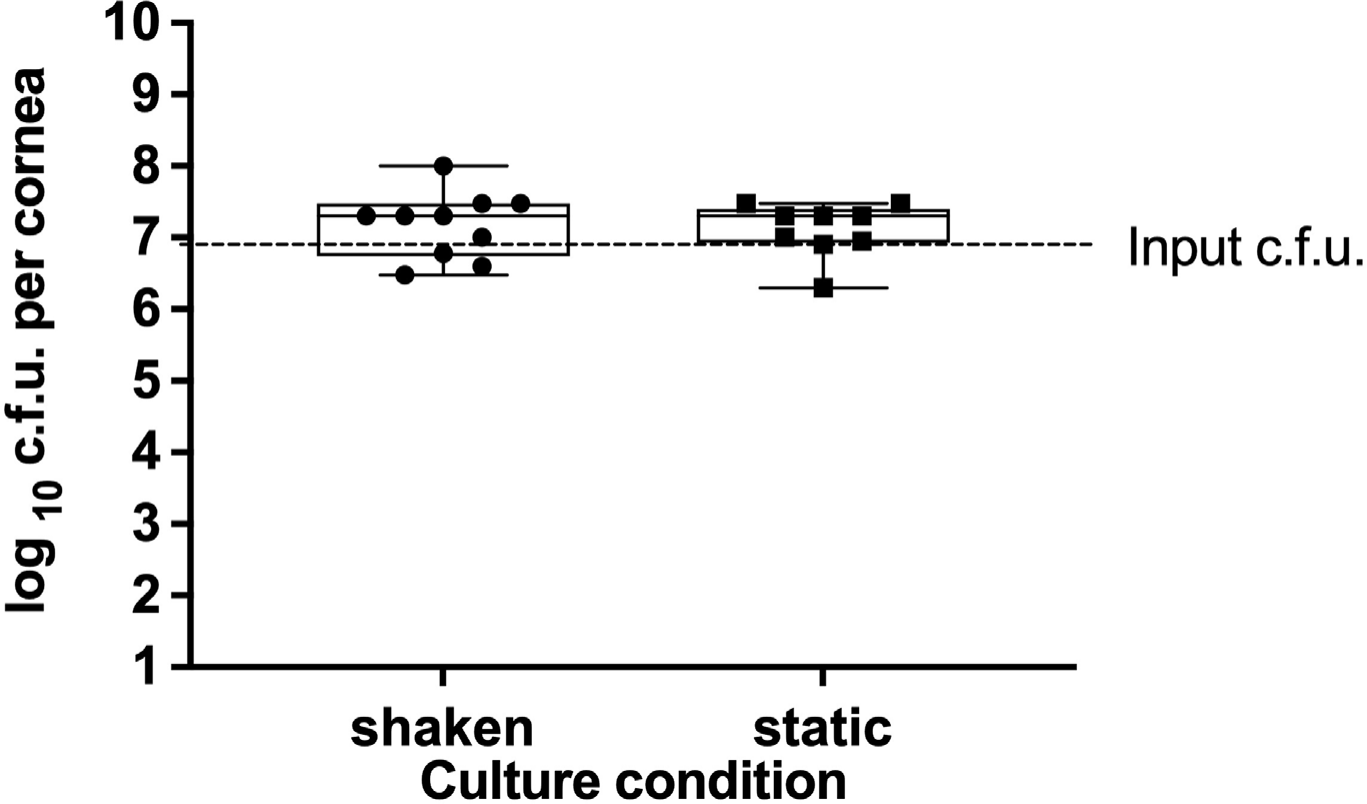
Examining the effect of rocking on infection of corneas. Comparing c.f.u. for *ex vivo* pig corneas incubated in DMEM/F12 without FBS and continuously rocked or cultured statically. The corneas were infected with *P. aeruginosa* PA01 for 6 h. The box plot shows the minimum and maximum values depicted by the bars. The top, middle and bottom horizontal lines demonstrate the upper, median and lower quartile. Statistical significance was calculated with the Mann–Whitney test, where a *P*-value<0.05 shows significance.

Finally, we aimed to determine whether rocking, which mimics the rinsing effect of tears on the corneal surface, plays a significant role in the progression of infection. *Ex vivo* pig corneas were maintained in DMEM/F12 media as described in the previous section but without adding FBS. A batch of corneas (*n*=10) was continuously shaken on the rocker in the incubator. At the same time, another set was cultured as per standard protocol, in static condition (*n*=9) until the infection (Fig. 8). Average colony counts were as follows: shaken 2×10^7^±3×10^7^, static 2×10^7^±1×10^7^. Rocking corneas had no significant impact (*P*>0.05) on the viable cell count after infecting for 6 h with *P. aeruginosa* PA01.

## Discussion

The corneas removed from donors degrade with time despite preservation efforts that affect their metabolic profile [17]; it is currently unknown if these changes affect infection outcomes in *ex vivo* keratitis models. This study aimed to explore the differences between *ex vivo* animal and human keratitis models and investigate if variations in culturing conditions and tissue maintenance affect the infection outcome. We compared c.f.u. in *ex vivo* human, pig and sheep corneas infected by *P. aeruginosa* PA01 using our previously published protocol [11]. The results of this research provide supporting evidence that differences such as storage temperature, time in storage, the composition of media and using different animal and human models have no significant effect on average c.f.u. retrieved from the corneas infected with 6×10^6^ c.f.u. of *P. aeruginosa* PA01 for 6 and 24 h.

When studying infection in *ex vivo* keratitis models, it is crucial to ensure that stratified squamous epithelium, the first barrier bacteria encounter, is well-preserved. Several factors affect the health and thickness of this epithelial layer in *ex vivo* corneas, which we briefly describe here. The thickness of the epithelial layer varies between species [14,18, 19], but despite these differences, the infection outcomes were similar across all the *ex vivo* models used in this study. Researchers have reported that the time between death and preservation can affect the quality of stratified squamous epithelium, particularly if it exceeds 6 h [20]. To maintain epithelial quality, we preserved all animal corneas used in this study within 6 h of harvesting. During storage, the corneas naturally begin to lose proteins [21], and epithelial cells undergo apoptosis [22,23]. However, studies found that only 13% of epithelial cells undergo apoptosis in human corneas stored *ex vivo* at 4 °C within 23 days of storage [24], suggesting that most of the stratified squamous epithelium remains metabolically active.

The healing process in *ex vivo* corneas [13,25] suggests that storing corneas in media for a few days before experiments may allow the stratified squamous epithelium to repair and recover. In live animals, most corneal epithelial cells are viable, with only a small percentage undergoing apoptosis [26]. However, the number of apoptotic cells can increase in response to dry eye [27], injury, trauma, exposure to irritants [28] or infection. Therefore, we anticipate enhanced epithelial shedding induced by cut trauma in our cornea models. We suspect that the lack of distinct differences between *ex vivo* models in our study may result from epithelial thinning during the infection with *P. aeruginosa* [29]. This bacterium not only attaches to damaged and apoptotic cells at the injury site [30] but also induces apoptosis through various mechanisms [31] and releases proteolytic enzymes or other toxic substances that reduce epithelial thickness [29]. These findings suggest that the stratified squamous epithelium, without the presence of a host defence system, is not a sufficient barrier to prevent the progression of the *P. aeruginosa* infection.

In our study, the stratified squamous epithelium was exposed to bacteria immersed in PBS during the infection. We chose PBS because its lack of nutrients forces bacteria to rely on the corneal epithelium for sustenance. Ideally, artificial tears would have been used to better mimic *in vivo* conditions, as the corneal epithelium depends on glucose and other nutrients present in tears for nourishment and maintenance [32], but they were not available. Tissue growth media, which contain much higher glucose levels than tears and many other nutrients not present in tears, could potentially alter bacterial growth. Exogenous glucose was found to promote the biofilm formation and antibiotic resistance in *P. aeruginosa* [33]. In contrast, artificial tears would have more accurately represented real-life conditions. *P. aeruginosa* also upregulates genes associated with bacterial survival and adaptation in response to human tear fluid [34]. However, it is unknown whether adding artificial tears could have led to a different infection outcome due to their effects on both *P. aeruginosa* and the corneal epithelium. Reduced nutrient availability, similar to our *ex vivo* keratitis model, can be observed in contact lens wearers or those with reduced tear production, potentially influencing infection severity [35]. Nevertheless, the use of PBS and maintaining all corneas in the same way resulted in no difference in c.f.u.s across animal and human models. All these factors need to be considered when interpreting the data from this study, and further research is necessary to fully understand these dynamics.

One of the culturing factors we investigated in this study was the storage temperature and duration before the infection procedure. Human corneas in tissue banks are typically stored at 34–37 °C (organ culture) for up to 4 weeks [36] or at 4 °C (hypothermia) for less than 2 weeks [24,37]. Both methods sufficiently preserve the integrity of endothelial and epithelial layers to ensure a successful graft transplantation [38]. While hypothermic storage at 4 °C slows down the metabolism and reduces the demand for energy, it can negatively impact the cornea, so it is recommended for storage periods of less than 2 weeks [24,37]. Long-term storage at 34^0^ C offers several advantages over shorter storage at 4 °C. It allows for the detection of corneas contaminated with micro-organisms and increases the availability of corneas for transplantation [36]. In our study, storage temperature did not affect the infection outcome in *ex vivo* human, pig and sheep keratitis models, suggesting that both storage methods are suitable. This is likely because all corneas were incubated in media for 3 days before infection, allowing them to equilibrate. Ensuring consistent tissue temperature is crucial, as temperature fluctuations can influence *P. aeruginosa* virulence factor expression [39] and potentially alter infection outcomes.

Prolonged storage of human corneas in media containing the broad spectrum aminoglycoside gentamicin could potentially slow bacterial penetration of the stratified squamous epithelium during the first few hours due to the accumulation of the antibiotic in the top epithelial layer. Gentamicin has been shown to accumulate in the cytosol of mammalian cells [40,41], but it binds to cell components and becomes microbiologically inactive [42]. Additionally, aminoglycosides can induce apoptotic cell death [43]. While the impact of long-term gentamicin exposure to mitochondrial damage in the corneal stratified squamous epithelium and its effect on *P. aeruginosa* infection remains uncertain, our results suggest that pre-infection storage of the corneas in different antibiotics does not alter the infection pattern. Washing with PBS and culturing corneas in antibiotic-free media for 48 h before infection was sufficient to eliminate any potential antimicrobial effects of residual antibiotics.

We observed that sheep and pig corneas started swelling after 2 days of incubation in the media. Swelling typically occurs after a trauma, injury, disease, infection or surgery and usually results from endothelial damage and changes in stromal hydration [44]. In contrast, human corneas did not swell as much as pig and sheep, which we suspect is related to the presence of Bowman’s layer. Bowman’s layer, which helps to maintain the cornea’s shape in humans [14], is either absent or much thinner in animals like pigs and sheep [15,16]. The structural difference is significant, as Bowman’s layer in humans has been shown to slow the progression of infection into the stroma [45]. However, in our experimental setup, the injury (cuts) to the cornea likely eliminates the protective benefit of this additional layer, allowing bacteria to reach the stroma. This may explain why there was no significant difference in infection progression between human and animal *ex vivo* keratitis models.

To prevent swelling, human corneas are typically stored in MK media supplemented with dextran [46], a supplement absent in the corneas from the NHSBT. We found that adding dextran to MK media reduced swelling in pig corneas, but there was no difference in viable counts between swollen and unswollen corneas after 24 h of incubation with *P. aeruginosa* PA01. Similarly, there was no difference in viable counts between human and animal models, suggesting that swelling does not interfere with or aid the infection progression. Further experiments confirmed that the presence or absence of dextran in media did not affect the infection outcome.

In terms of media composition, we ensured that epidermal growth factor and insulin were included, as these compounds have been shown to preserve corneal metabolic functions [47]. A different media composition (MK, EMEM and DMEM) during initial storage and the addition of FBS did not affect the infection outcome in *ex vivo* keratitis models. FBS is commonly used to support cell growth, attachment and proliferation *in vitro* [48]; however, its use raises ethical concerns due to the suffering caused by its extraction from living calf foetuses [49]. Our experiments showed that FBS did not impact infection outcomes, likely because the corneas were rinsed thoroughly with PBS before the infection procedure. This suggests that FBS can be removed from the media, offering an ethical advantage for our *ex vivo* keratitis model. However, the benefit of using FBS in maintaining *ex vivo* corneal epithelium should be investigated further before its complete elimination.

Additionally, while some studies suggest that air–liquid culturing with rocking corneas gives superior corneal tissue preservation compared to static, continuously immersed tissue [13], and reduces cellular apoptosis [50], we found no differences in c.f.u. between corneas maintained in air–liquid (rocked) and submerged (static) cultures before infection. Our data suggest that both methods are suitable for infection studies. However, although rocking and static conditions did not affect the final c.f.u., it is unknown to what extent they may influence bacterial attachment, infection progression and biofilm development. Rocking conditions partially mimic the *in vivo* scenario of blinking and tear rinsing, which could impact these factors. Therefore, these conditions may need further exploration, and the results should be interpreted with caution.

The present study has several limitations, particularly regarding donor variability. Human corneas varied in age, gender, ethnicity and overall donor’s health, introducing potential biases that were not present in the more uniform sheep and pig corneas, which were roughly the same breed and age. Additionally, the handling procedures differed: animal corneas were harvested and processed within 2 to 4 h postmortem and transferred to media immediately after enucleation, whereas the time between death and processing was likely longer for human corneas. The lack of histology images for human corneas prevented us from assessing the thickness and quality of epithelial surfaces in these samples.

Another factor is the variation in experimental conditions. For example, when human corneas were processed in India, the higher ambient temperature in the laboratory could have accelerated bacterial proliferation, potentially affecting c.f.u. counts. In contrast, human corneas from the NHSBT, as well as pig and sheep corneas, were processed in the same laboratory under identical conditions, minimizing variation. All corneas were infected using a previously published protocol, with the glass mould consistently applied to each species.

To strengthen the rigour of the study, we ensured that all corneas were processed within the same time interval and in the same laboratory, using the same equipment and batch of FBS across all experiments. However, the scarcity of human corneas for research limited the number of replicates, which is a natural drawback of this study. The initial handling and age of human corneas were beyond our control and could contribute to variability between replicates.

We faced challenges in securing a plentiful supply of sheep eyes, whereas pig eyes were more readily available, cheaper and easier to process. Consequently, pig corneas were used to test media composition and the effect of rocking. While we demonstrated that the infection pattern in *ex vivo* models is consistent across various animals and humans, we cannot exclude potential differences in infection on a molecular level. *Ex vivo* keratitis models also lack host immune responses and the presence of tears, which is another limitation. Despite these drawbacks, these models provide valuable insights into the interaction between bacteria and tissue during infection. Future studies may explore the addition of tears to further enhance the relevance of these models.

The main conclusion from this study is that the patterns in infection with *P. aeruginosa* between *ex vivo* pig and sheep keratitis models match humans. Culture conditions and the addition of dextran did not affect the infection outcome in our *ex vivo* keratitis models. The media for *ex vivo* keratitis models can be free of animal components which adds the benefit of following Replacement, Reduction and Refinement (3Rs) principles. In many facilities, the corneas of a specific species may not be available to establish the *ex vivo* model. Consequently, other species, such as sheep, pigs or rabbits, can be used. The consistent results are attributed to the use of our glass mould, which was effective for sheep, porcine and human corneas, demonstrating its broad applicability across species. Furthermore, this experiment adds to a growing corpus of research showing the benefits and potential of *ex vivo* keratitis models in infection studies.

## Abbreviations

DMEM: Dulbecco’s Modified Eagle’s Medium
EGF: epidermal growth factor
EMEM: Eagle’s Minimum Essential Medium
FBS: foetal bovine serum
H&E: haematoxylin and eosin
LVPEI: LV Prasad Eye Institute
MK: McCarey-Kaufman
NHSBT: National Health Service Blood and Transplant

## References

[1] Whitcher JP, Srinivasan M, Upadhyay MP (2001). Corneal blindness: a global perspective. Bull World Health Organ.

[2] Dave A, Samarth A, Karolia R, Sharma S, Karunakaran E (2020). Characterization of ocular clinical isolates of *Pseudomonas aeruginosa* from non-contact lens related keratitis patients from south India. Microorganisms.

[3] Årdal C, Balasegaram M, Laxminarayan R, McAdams D, Outterson K (2020). Antibiotic development - economic, regulatory and societal challenges. Nat Rev Microbiol.

[4] Langdon A, Crook N, Dantas G (2016). The effects of antibiotics on the microbiome throughout development and alternative approaches for therapeutic modulation. Genome Med.

[5] Payne DJ, Gwynn MN, Holmes DJ, Pompliano DL (2007). Drugs for bad bugs: confronting the challenges of antibacterial discovery. Nat Rev Drug Discov.

[6] Urwin L, Okurowska K, Crowther G, Roy S, Garg P (2020). Corneal infection models: tools to investigate the role of biofilms in bacterial keratitis. Cells.

[7] Madhu SN, Jha KK, Karthyayani AP, Gajjar DU (2018). *Ex vivo* caprine model to study virulence factors in keratitis. *J Ophthalmic Vis Res*.

[8] Pinnock A, Shivshetty N, Roy S, Rimmer S, Douglas I (2017). Ex vivo rabbit and human corneas as models for bacterial and fungal keratitis. *Graefes Arch Clin Exp Ophthalmol*.

[9] Sullivan AB, Tam KPC, Metruccio MME, Evans DJ, Fleiszig SMJ (2015). The importance of the *Pseudomonas aeruginosa* type III secretion system in epithelium traversal depends upon conditions of host susceptibility. Infect Immun.

[10] Okurowska K, Monk PN, Karunakaran E (2024). Increased tolerance to commonly used antibiotics in a *Pseudomonas aeruginosa ex vivo* porcine keratitis model. Microbiology.

[11] Okurowska K, Roy S, Thokala P, Partridge L, Garg P (2020). Establishing a porcine *ex vivo* cornea model for studying drug treatments against bacterial keratitis. J Vis Exp.

[12] Russell WMS (1999). The progress of humane experimental technique. *Altern Lab Anim*.

[13] Deshpande P, Ortega I, Sefat F, Sangwan VS, Green N (2015). Rocking media over ex vivo corneas improves this model and allows the study of the effect of proinflammatory cytokines on wound healing. Investig Ophthalmol Visual Sci.

[14] Sridhar MS (2018). Anatomy of cornea and ocular surface. Indian J Ophthalmol.

[15] Batista A, Breunig HG, Uchugonova A, Morgado AM, König K (2016). Two-photon spectral fluorescence lifetime and second-harmonic generation imaging of the porcine cornea with a 12-femtosecond laser microscope. J Biomed Opt.

[16] Crespo-Moral M, García-Posadas L, López-García A, Diebold Y (2020). Histological and immunohistochemical characterization of the porcine ocular surface. PLoS One.

[17] Kryczka T, Ehlers N, Nielsen K, Midelfart A (2012). Impact of organ culturing on metabolic profile of human corneas: preliminary results. Acta Ophthalmol.

[18] Jay L, Brocas A, Singh K, Kieffer JC, Brunette I (2008). Determination of porcine corneal layers with high spatial resolution by simultaneous second and third harmonic generation microscopy. Opt Express.

[19] LoPinto AJ, Pirie CG, Bedenice D, Ayres SL (2017). Corneal thickness of eyes of healthy goats, sheep, and alpacas manually measured by use of a portable spectral-domain optical coherence tomography device. Am J Vet Res.

[20] Kim BJ, Sprehe N, Morganti A, Wordinger RJ, Clark AF (2013). The effect of postmortem time on the RNA quality of human ocular tissues. Mol Vis.

[21] Hasany SM, Basu PK (1987). Changes of MK medium during storage of human cornea. Br J Ophthalmol.

[22] Albon J, Tullo AB, Aktar S, Boulton ME (2000). Apoptosis in the endothelium of human corneas for transplantation. Investig Ophthalmol Visual Sci.

[23] Crewe JM, Armitage WJ (2001). Integrity of epithelium and endothelium in organ-cultured human corneas. Investig Ophthalmol Visual Sci.

[24] Komuro A, Hodge DO, Gores GJ, Bourne WM (1999). Cell death during corneal storage at 4 degrees C. Investig Ophthalmol Vis Sci.

[25] Castro N, Gillespie SR, Bernstein AM (2019). *Ex vivo* corneal organ culture model for wound healing studies. JoVE.

[26] Ren HW, Wilson G (1996). Apoptosis in the corneal epithelium. Investig Ophthalmol Visual Sci.

[27] Yeh S, Song XJ, Farley W, Li DQ, Stern ME (2003). Apoptosis of ocular surface cells in experimentally induced dry eye. Invest Ophthalmol Vis Sci.

[28] Ambrósio R, Kara-José N, Wilson SE (2009). Early keratocyte apoptosis after epithelial scrape injury in the human cornea. Exp Eye Res.

[29] Marquart ME, O’Callaghan RJ (2013). Infectious keratitis: secreted bacterial proteins that mediate corneal damage. J Ophthalmol.

[30] Capasso D, Pepe MV, Rossello J, Lepanto P, Arias P (2016). Elimination of *Pseudomonas aeruginosa* through efferocytosis upon binding to apoptotic cells. PLoS Pathog.

[31] Kaminski A, Gupta KH, Goldufsky JW, Lee HW, Gupta V (2018). *Pseudomonas aeruginosa* ExoS induces intrinsic apoptosis in target host cells in a manner that is dependent on its GAP domain activity. Sci Rep.

[32] Lam SM, Tong L, Duan X, Petznick A, Wenk MR (2014). Extensive characterization of human tear fluid collected using different techniques unravels the presence of novel lipid amphiphiles. J Lipid Res.

[33] She P, Wang Y, Liu Y, Tan F, Chen L (2019). Effects of exogenous glucose on *Pseudomonas aeruginosa* biofilm formation and antibiotic resistance. Microbiologyopen.

[34] Tabor LM, Grosser MR, Metruccio MMME, Kumar NG, Wu YT (2021). Human tear fluid modulates the *Pseudomonas aeruginosa* transcriptome to alter antibiotic susceptibility. Ocul Surf.

[35] Gurnani B, Kaur K (2023). Contact lens-related complications. https://www.ncbi.nlm.nih.gov/books/NBK587443/.

[36] Armitage WJ (2011). Preservation of human cornea. Transfus Med Hemother.

[37] Hsu JKW, Cavanagh HD, Jester JV, Ma LS, Petroll WM (1999). Changes in corneal endothelial apical junctional protein organization after corneal cold storage. Cornea.

[38] Ehlers H, Ehlers N, Hjortdal JO (1999). Corneal transplantation with donor tissue kept in organ culture for 7 weeks. Acta Ophthalmol Scand.

[39] Roncarati D, Vannini A, Scarlato V (2024). Temperature sensing and virulence regulation in pathogenic bacteria. Trends Microbiol.

[40] Hendrix DVH, Ward DA, Barnhill MA (2001). Effects of antibiotics on morphologic characteristics and migration of canine corneal epithelial cells in tissue culture. Am J Vet Res.

[41] Imbuluzqueta E, Lemaire S, Gamazo C, Elizondo E, Ventosa N (2012). Cellular pharmacokinetics and intracellular activity against *Listeria monocytogenes* and *Staphylococcus aureus* of chemically modified and nanoencapsulated gentamicin. J Antimicrob Chemother.

[42] Nix DE, Goodwin SD, Peloquin CA, Rotella DL, Schentag JJ (1991). Antibiotic tissue penetration and its relevance: impact of tissue penetration on infection response. Antimicrob Agents Chemother.

[43] O’Reilly M, Young L, Kirkwood NK, Richardson GP, Kros CJ (2019). Gentamicin affects the bioenergetics of isolated mitochondria and collapses the mitochondrial membrane potential in cochlear sensory hair cells. Front Cell Neurosci.

[44] Wilson SE, Sampaio LP, Shiju TM, Hilgert GSL, de Oliveira RC (2022). Corneal opacity: cell biological determinants of the transition from transparency to transient haze to scarring fibrosis, and resolution, after injury. Invest Ophthalmol Vis Sci.

[45] Alarcon I, Kwan L, Yu C, Evans DJ, Fleiszig SMJ (2009). Role of the corneal epithelial basement membrane in ocular defense against *Pseudomonas aeruginosa*. Infect Immun.

[46] Abdin A, Daas L, Pattmöller M, Suffo S, Langenbucher A (2018). Negative impact of dextran in organ culture media for pre-stripped tissue preservation on DMEK (Descemet membrane endothelial keratoplasty) outcome. *Graefes Arch Clin Exp Ophthalmol*.

[47] Lass JH, Putman SC, Bruner WE, Cano DB, Greiner JV (1994). The effect of hEGF and insulin on corneal metabolism during Optisol storage. Cornea.

[48] Gstraunthaler G, Lindl T, van der Valk J (2013). A plea to reduce or replace fetal bovine serum in cell culture media. Cytotechnology.

[49] Jochems CEA, van der Valk JBF, Stafleu FR, Baumans V (2002). The use of fetal bovine serum: ethical or scientific problem?. *Altern Lab Anim*.

[50] Marlo TL, Giuliano EA, Sharma A, Mohan RR (2017). Development of a novel ex vivo equine corneal model. Vet Ophthalmol.

